# Appropriate ICD Interventions for Ventricular Arrhythmias Are Predicted by Higher Syntax Scores I and II in Patients with Ischemic Heart Disease

**DOI:** 10.3390/jcm10091843

**Published:** 2021-04-23

**Authors:** Teresa Strisciuglio, Giuseppe Ammirati, Valerio Pergola, Lucio Addeo, Maria Angela Losi, Aniello Viggiano, Livio Imparato, Vincenzo Russo, Enrico Melillo, Gerardo Nigro, Giuseppe Stabile, Antonio D’Onofrio, Giovanni Esposito, Antonio Rapacciuolo

**Affiliations:** 1 Department of Advanced Biomedical Sciences, University of Naples Federico II, 80131 Naples, Italy; teresa.strisciuglio@unina.it (T.S.); giuseppe.ammirati92@gmail.com (G.A.); valerio.pergola.pz@gmail.com (V.P.); addeolucio@gmail.com (L.A.); mariaangela.losi@unina.it (M.A.L.); aniello.viggiano@unina.it (A.V.); livioimparato91@gmail.com (L.I.); espogiov@unina.it (G.E.); 2 Department of Medical Translational Sciences, University of Campania “Luigi Vanvitelli”, 80131 Naples, Italy; v.p.russo@libero.it (V.R.); e.melillo@hotmail.fr (E.M.); gerardo.nigro@unicampania.it (G.N.); donofrioant1@gmail.com (A.D.); 3 Department of Cardiology, Clinica Montervergine, 83013 Mercogliano, Italy; gmrstabile@tin.it

**Keywords:** ventricular arrhythmias, coronary artery disease, implantable cardiac defibrillator, heart failure

## Abstract

Aims. The occurrence of ventricular arrhythmias (VAs) in ischemic heart disease (IHD) patients is related to the presence and extent of fibrotic/scar tissue. As coronary atherosclerosis is the underlying cause of myocardial ischemia and fibrosis, in IHD patients implanted with an implantable cardioverter defibrillator (ICD) we investigated the relation between the VA burden and the complexity of coronary atherosclerotic lesions. Methods and results. In IHD patients who underwent coronary angiography and ICD implant, the Syntax scores I and II (SSI-II), as index of the severity of the coronary atherosclerotic disease, and the occurrence of VA were assessed. Overall 144 patients were included (123 males). Of these 22 patients (15%) experienced at least one episode of VA (cycle length 298 ± 19 msec) that required ICD intervention. The number of episodes per patient and per year was 4 ± 6 and 2.8 ± 4, respectively. Patients that experienced a VA compared to those free from arrhythmic events did not have distinct baseline clinical characteristics except for a higher SS I and SS II (21 (IQR 13–38) vs. 16 (IQR 10–23); *p* = 0.037; and 50 (IQR 39–62) vs. 42 (IQR 34–50); *p* = 0.012). In the binary logistic regression analyses the SS I and II were the only independent predictors of VA occurrence. A higher SS II was also associated with an earlier time to first event (*p* = 0.005). Conclusion. Higher SS I-II scores reflect a more severe coronary atherosclerosis and are associated with a greater VA burden. Further studies are needed to better clarify the ability of SSI-II to stratify the risk of IHD patients to develop life-threatening VA.

## 1. Introduction

The implantable cardioverter defibrillator (ICD) is recommended for primary prevention of sudden cardiac death (SCD) in patients with ischemic heart disease (IHD) and dilated cardiomyopathy associated with severe reduction of the left ventricular ejection fraction (LVEF) (≤35%) [1].

However, up to 75–80% of patients remain free of appropriate ICD therapy during the lifetime of their first ICD generator [2].

The ventricular fibrosis, generated by myocardial ischemia, leads to electrical conduction anisotropy, which represents the substrate for re-entrant circuits, thus for ventricular arrhythmias (VAs) [3]. The amount and the trans-mural extent of the fibrosis measured by late gadolinium enhancement cardiovascular magnetic resonance (LGE-CMR) is associated with the burden of VA [4] and might be a stronger predictor of outcomes than the LVEF [5]. Although LGE-CMR is a valid risk stratification tool for the occurrence of ventricular arrhythmias, nonetheless its availability and costs may represent a limitation for its use in the daily practice, therefore new stratification tools are needed to help the patient selection for ICD therapy.

In IHD patients the extent of myocardial fibrosis depends on the underlying ischemia related to the severity of coronary atherosclerosis. We investigated whether the Syntax score I (SSI) [6] and the Syntax score II (SSII) [7], which are scores widely used to grade the complexity of atherosclerotic lesions, are associated with the ventricular arrhythmic burden.

## 2. Methods

### 2.1. Patients’ Enrolment

Consecutive patients with IHD implanted with an ICD in two centers (University of Naples Federico II and University of Campania “Luigi Vanvitelli) for reduced LVEF (≤35%) were included if they underwent a coronary angiography (CA) no longer than 3 months before the implant. After the revascularization patients with residual intermediate stenoses at the CA underwent a cardiac single-photon emission computerized tomography (SPECT) to exclude the presence of residual ischemia. Patients were included in the study if they had a minimum follow-up of 12 months. All patients signed the informed consent for data collection and the local Ethics Committee Carlo Romano of the University of Naples Federico II approved the protocol (project identification code 159/15). The study is in accordance with the Declaration of Helsinki.

### 2.2. Assessment of Ventricular Arrhythmic Burden and Clinical Management

**Figure 1:**
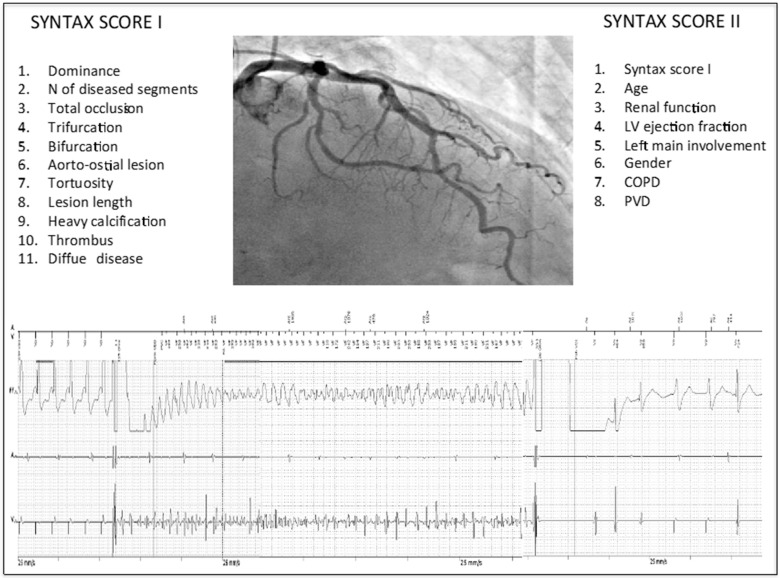
Assessment of SSI and SS II by looking at cine-loops of coronary angiography (**upper panel**). Evaluation and confirmation of each VT/VF episodes registered by the ICD (**lower panel**).

The ICD interrogation was performed every 6 months after the implant during outpatient visits. The ventricular tachycardia/fibrillation (VT/VF) episodes, either sustained or not, reported by the device were checked by two independent electrophysiologists (Figure 1), and the false episodes misinterpreted by the device (e.g., atrial fibrillation with high ventricular response, aberrant conduction, and artifacts) were discarded. The true sustained VT/VF episodes that required an appropriate antitachycardia pacing (ATP) or shock and their characteristics were collected in a local database. The decisions regarding the antiarrhythmic therapy, including drug replacement or dosage increase/reduction, were left to the electrophysiologists’ discretion. The electrophysiologists were blinded to the extent of coronary atherosclerosis of the patients.

### 2.3. Assessment of Coronary Atherosclerosis

Assessment of the coronary atherosclerosis was performed by two interventional cardiologists at the moment of the coronary angiography and after percutaneous revascularization. The SS I was used as an index of the severity of coronary atherosclerosis and was calculated retrospectively from the cine-loops of the CA by two independent interventional cardiologists (Figure 1). This score takes into account 11 variables: Dominance, number of diseased segments, total occlusion, trifurcation, bifurcation type and angulation, aorto-ostial lesion, tortuosity, lesion length, heavy calcification, thrombus, and diffuse disease [6].

The SS II score considers both the extension and the severity of coronary atherosclerosis (SS I), and clinical variables as age, renal function (creatinine clearance), LVEF, left main involvement, sex, presence of chronic obstructive pulmonary disease (COPD), and of peripheral vascular disease (PVD) [7]. Either scores were easily calculated via a web calculator. When the SS differed between the cardiologists they had to reach a consensus by reviewing together the cine-loop recordings. The extent of coronary atherosclerosis was measured based on the number of coronary vessel having significant atherosclerotic lesions ≥50%. The interventional cardiologists were blinded to the arrhythmic burden of the patients.

### 2.4. Statistics

Descriptive statistics are reported as means ± SD for normally distributed continuous variables, or medians with 25th to 75th percentiles in the case of skewed distribution. Normality of distribution was tested by means of the nonparametric Kolmogorov–Smirnov test. Differences between mean data were compared by means of a *t*-test for Gaussian variables, and the F-test was used to check the hypothesis of equality of variance. The Mann–Whitney non-parametric test was used to compare non-Gaussian variables. The differences in proportions were tested by applying χ^2^ analysis or Fisher’s exact test, as appropriate. Intraobserver and interobserver variability of SYNTAX I and SYNTAX II were evaluated using intraclass correlation (ICC) and their 95% confident intervals, based on a mean rating (k = 2), absolute-agreement, 2-way mixed-effects model. Univariable and multivariable logistic regression analyses were performed to identify the predictors of VT/VF occurrence.

In the univariate analysis, every effect was considered separately in a single model. In multivariable analysis, relevant characteristics were considered together. Multivariable models were developed starting from variables with *p* < 0.2 in the univariable analysis and then implementing a stepwise backward selection, comparing the likelihood of the data under the full model against the likelihood of the data under a model with fewer predictors, to include all suited factors with better fit the models. All interactions between the covariates were analyzed. Results of regression analyses were presented as odds ratio (OR) (95% IC). The optimal diagnostic cut-off value of SS II was defined based on the Youden’s index, calculated as ((sensitivity + specificity) − 1), namely where the sum of sensitivity and specificity is maximized. Statistical significance was set at a 2-tailed probability level of <0.05. All statistical analyses were performed by means of SPSS 25.0 (IBM, Armonk, New York, NY, USA).

## 3. Results

### 3.1. Patients’ Population

One hundred and forty-four patients were included, of these 123 (72%) were males and the mean LVEF was 30% ± 4%. Baseline clinical characteristics are reported in Table 1. Twelve patients were implanted with a single-chamber ICD, 89 with a dual-chamber, 31 with a CRT-D, and 12 had a device up-grade from single-chamber ICD. Twenty-two patients (15%) experienced at least one episode of VT/VF that required an appropriate ATP/shock. No significant differences in clinical characteristics neither in LVEF were found between patients that experienced or not a VT/VF episode (Table 1). Patients who experienced a VF episode had no distinct clinical and/or angiographic characteristics compared to those who experienced a VT episode. The prevalence of atrial fibrillation (paroxysmal/permanent) was not higher in patients experiencing a ventricular event. Furthermore, patients that experienced at least one VT/VF episode were more likely to take antiarrhythmic drugs (*p* = 0.013). In the overall population the median SS I was 17 (IQR 10–26) and the median SS II was 42 (IQR 34–52). The interrater and intrarater reliability was good (95%-confidence interval for ICC population values: 0.91–0.983, *p* value < 0.0001).

### 3.2. VT/VF Episodes and Their Treatment

In the 22 patients having a VT//VF episode the overall number of events was 90 (8 VF, 28 VT in the VF window, and 54 VT) with a median cycle length of 298 ± 19 msec. The number of episodes per patient was 4 ± 6 and the number of episodes per year was 2.8 ± 4. These events were treated in 66% of cases with ATP, in 18% with DC-shock, and in 16% with unsuccessful ATP followed by DC-shock.

### 3.3. Association between the Occurrence of Ventricular Events and Coronary Atherosclerosis

**Figure 2:**
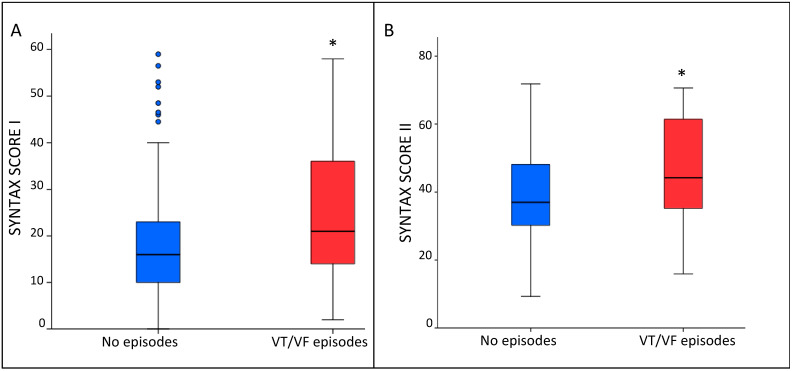
Whisker plots showing the relation between SS I (**A**) and SS II (**B**) in patients who experienced VT/VF episodes and in those who did not. The symbol “*” represents a significant *p* value < 0.05; the symbol “°” represents an outlier.

In patients in whom at least one episode occurred the SS I and SS II were significantly higher compared to patients that didn’t experience any episode (21 (IQR 13–38) vs. 16 (IQR 10–23); *p* = 0.037; and 50 (IQR 39–62) vs. 42 (IQR 34–50); *p* = 0.012) (Figure 2 panel A and B, respectively).

Univariate analysis showed that SS I and II and the treatment with amiodarone were related with the occurrence of VT/VF events (Table 2). No significant interactions were observed between the SS I and II and all the other covariates except for the SS I and the coronary revascularization (*p* = 0.0042). The subgroup analysis revealed that in patients underwent revascularization the SS I was the only independent predictor of ventricular arrhythmic events OR 1.09 (1.03–1.15) *p* = 0.002 at the multivariate binary logistic regression analysis, whereas the SS I was not significant in the overall population (Table 2).

As for the SS II, this was an independent predictor of ventricular arrhythmic events and the amiodarone intake (Table 2).

Furthermore, based on the ROC curve the best value of SS II that predicted the occurrence of VT/VF was 52 or above (area under the curve AUC = 0.64) (sensitivity 45% and specificity 80%).

### 3.4. Syntax Score II and Time to First Ventricular Arrhythmic Event

**Figure 3:**
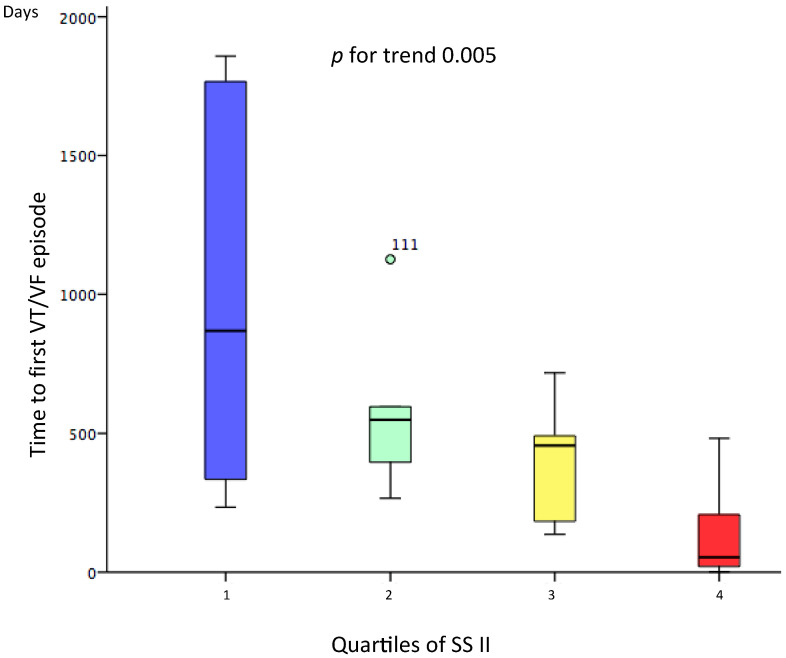
Whisker plots showing the time to recurrence of the VT/VF episode in the different quartiles of the SS II. The symbols ° represents an outlier.

The median follow up was 1340 days (IQR 799–2012). In the 22 patients with episodes of VT/VF the median time to first event was 334 (62–1048) days. According to the distribution in quartiles of SSII, those patients in the highest quartile had a shorter time to first event (*p* for trend = 0.005) (Figure 3).

## 4. Discussion

### 4.1. Main Findings

In the present study we highlighted in ischemic patients the association between the ventricular arrhythmic burden and the complexity and severity of coronary atherosclerosis as patients with at least one episode of VT/VF treated with ATP/shock by the ICD had higher SS I and SS II. SS II is an independent predictor of the occurrence of ventricular events. Patients with higher SS II have a shorter time to the first event.

### 4.2. Predictors of Appropriate ICD Interventions in Ischemic Patients

Patients with IHD implanted with an ICD in primary or secondary prevention may experience or not VA requiring therapy during the battery lifespan of the device [2]. As the healthcare resources may be limited and some complications may occur during generator replacement, it is important to identify those patients who really need it, thus so far a great attention has been paid to the predictors of the occurrence of ventricular arrhythmic events.

Witt et al. demonstrated that between initial ICD implantation and first generator replacement at least 1 appropriate ICD therapy for ventricular arrhythmia occurred in about 33.1% of patients after a mean time of 1.9 ± 1.8 years in patients with IHD [8]. Similarly in a CRTD population, Friedman et al. demonstrated that the 4-year incidence of appropriate device therapy was 36% and furthermore those with LVESD > 61 mm had a 51% 3-year incidence of VA. Other predictors of arrhythmic events were absence of beta-blocker therapy, LVEF < 20% and history of sustained VA [9]. Furthermore, in the work by Verma et al. predictors of appropriate ICD therapy were hypertension and non-sustained episodes of VT whereas EF, NYHA class and QRS width were not [10]. Conversely, the PROFIT study demonstrated that a QRS ≥ 150 msec was an independent predictor of VT/VF occurrence in ICD patients together with a low LVEF and with permanent atrial fibrillation (AF) [11]. The link between AF and VA was also confirmed in a recent study by Vergara et al. who found in a cohort of 2435 ICD/CRTD patients that atrial high rate episodes (AHREs) were associated with increased risk of a ventricular arrhythmic event and with increased mortality [12].

### 4.3. Occurrence of VT/VF in Ischemic Patients

A severe CAD may cause myocardial ischemia that if prolonged leads to the death of cardiac myocytes and to the replacement with fibrotic tissue. This fibrosis either intra- or transmural affects ventricular depolarization, as the surviving bundles of myocytes within the infarct scar form slow-conduction channels. The anisotropy of conduction of the electrical impulse predisposes to re-entrant mechanisms that result in the occurrence of post-infarct monomorphic VT [13].

The extent of myocardial scar, either the overall percentage of scar tissue either the number of transmural scar segments, characterized by LGE-CMR is significantly associated with the occurrence of spontaneous VA [4]. Moreover, Wu et al. demonstrated that in a population o STEMI patients acute infarct size obtained by contrast enhanced CMR was a stronger predictor of outcomes (death, recurrent MI and heart failure) than LVEF and end-systolic left ventricular volume [5].

Nonetheless there are also less expensive and more accessible risk stratification tools.

The not uniform ventricular depolarization related to myocardial scar can be identified in some cases by the presence of additional spikes within the QRS complexes on the electrocardiogram (fragmented QRS, fQRS). The presence of fQRS complex on ECG was found to be associated with the occurrence of in-hospital life-threatening arrhythmic events in STEMI patients [14] and with the severity of CAD (Syntax Score) in NSTEMI patients [15]. In addition, in the INFUSE AMI trial, Redfors et al. demonstrated that in STEMI patients undergoing primary PCI, those having fQRS had poorer outcomes (death, target vessel revascularization, and target vessel myocardial infarction) but the infarct size was similar compared to patients without fQRS [16]. Therefore the prognostic value of fQRS is still a matter of debate.

Our study demonstrates for the first time that the ventricular arrhythmic burden in ICD patients is associated with the SS I and II, and especially patients with higher SS II experience an earlier VT/VF episode. These findings corroborate the tight link between coronary artery disease, scar and the ventricular arrhythmic burden.

### 4.4. Conclusions

The SSI/SSII may be a valuable stratification tool for the selection of IHD patients with higher VT/VF burden who may benefit more of an ICD implantation/replacement.

As the SS/SSII measure the complexity of coronary atherosclerosis we can speculate that it partially reflects the subendocardial/transmural myocardial fibrosis/scar, which is the substrate of re-entrant VT. More studies are needed to better clarify the ability of SSI and SSII to stratify the risk of IHD patients to develop life-threatening ventricular arrhythmias. In fact, considering only LVEF can be considered a rough method of patients’ stratification. Therefore, a multiparametric approach is definitely desirable.

#### Study Limitations

This study had several limitations. First, the small sample size weakened our results and further larger studies are needed to corroborate our novel findings. However the patients’ number is similar to previous studies reporting on this subject. Second, a CMR to assess endocardial and/or transmural fibrosis was not performed, therefore we could only hypothesize that higher SSI-SSII could be associated with increased fibrosis. Third, we could not exclude some bias related to the differences in antiarrhythmic drug however any influence of the treatment on the occurrence of VA was excluded at multivariable analysis. Fourth, we did not investigate other variables that have been demonstrated to be predictors of VT/VF occurrence. Fifth, the device setting for VT/VF detection were not pre-specified, due to the retrospective nature of the study. This is a hypothesis-generating study and further prospective and randomized studies are needed to confirm the role of SS as stratification tool for ventricular arrhythmic events.

## Figures and Tables

**Table 1:** Baseline clinical characteristics.

Variables	Overall (*n* = 144)	No Events (*n* = 122)	V Events (*n* = 22)	*p*
Males, *n* (%)	123 (85.4)	102 (84)	21 (95)	0.2
Age, median (IQR)	64 (55–72)	63 (55–72)	68 (56–80)	0.5
Diabetes, *n* (%)	67 (46.5)	57 (47)	10 (46)	1
Hypertension, *n* (%)	113 (78.5)	95 (84)	18 (82)	0.8
Smoke, *n* (%)	74 (51.4)	64 (53)	10 (46)	0.6
Dyslipidemia, *n* (%)	101 (70.1)	83 (68)	18 (82)	0.3
Revascularization, *n* (%)	77 (53,5%)	63 (81.8)	14 (18.2)	<0.001
CKD, *n* (%)	44 (30.6)	35 (29)	9 (41)	0.3
AF *n* (%)	30 (20.8)	25 (21)	5 (23)	0.8
Pre-EF, %	30 (28–33)	30 (28–33)	30 (30–35)	1
BBs, *n* (%)	98 (72.1)	84 (73)	14 (67)	0.6
ACEIs	78 (54)	63 (52)	15 (68)	0.4
ARBs	33 (23)	30 (25)	3 (14)	0.4
Amiodarone, *n* (%)	27 (19)	18 (15)	9 (41)	0.013

CKD indicates chronic kidney disease; AF indicates atrial fibrillation; pre-EF indicates ejection fraction predevice implant; BBs indicates beta-blockers; BBs indicates Beta blockers; ACEIs indicates angiotensin converting enzyme inhibitors; ARBs indicates Angiotensin II receptor blockers.

**Table 2:** Univariate analysis and multivariate analysis 1 (MV1) including SS I and multivariate analysis 2 (MV2) including SS II. Statistical significance between VT/VF episodes occurrence and SS II was always observed even after adjustment for all the covariates; panel C shows the best fitting regression model.

Univariate.	OR (95% CI)	*p* Value
Gender	4.1 (0.5–32.3)	0.18
Age	1.04 (0.99–1.08)	0.82
Hypertension	1.28 (0.39–4.1)	0.68
Smoke	0.75 (0.304–1.87)	0.54
Diabetes	0.95 (0.38–2.4)	0.9
Dyslipidemia	2.1 (0.67–6.6)	0.2
Revascularization	1.63 (0.65–4.36)	0.3
Atrial fibrillation	1.1 (0.38–3.4)	0.8
PVD	2.12 (0.85–5.32)	0.107
Pre-EF	1.05 (0.93–1.17)	0.4
CKD	1.7 (0.67–4.4)	0.26
Amiodarone	3.88 (1.44–10.42)	0.007
BBs	0.73 (0.27–1.99)	0.55
SYNTAX I	1.04 (1.004–1.07)	0.029
SYNTAX II	1.05 (1.01–1.09)	0.015
**MV1**	**OR (95% CI)**	***p* Value**
Gender	4.63 (0.73–94.7)	0.17
PVD	1.7 (0.6–4.6)	0.32
Dyslipidemia	2.5 (0.77–9.9)	0.15
Revascularization	1.74 (0.64-5)	0.28
Amiodarone	4.76 (16–14.3)	0.04
SS I	1.03 (0.09–1.06)	0.09
**MV2**	**OR (95% CI)**	***p* Value**
Dyslipidemia	3.2 (0.92–15.6)	0.09
Beta-Blockers	1.1 (0.37–3.8)	0.83
Amiodarone	5.06 (1.7–15.6)	0.03
SS II	1.06 (1.01–1.12)	0.009

CKD indicates chronic kidney disease; AF indicates atrial fibrillation; pre-EF indicates ejection fraction pre-device implant; SS I indicates Syntax score I; SS II indicates Syntax score II; OR indicates odds ratio; CI indicates confidence intervals.

## Data Availability

Data are available upon reasonable request to the correspondent author.
